# The Diagnostic Value of *β*-Human Chorionic Gonadotropin, Progesterone, and Ischemia-Modified Albumin and Their Combined Use in the Prediction of First Trimester Abortions

**DOI:** 10.1155/2014/846531

**Published:** 2014-07-01

**Authors:** Mehmet A. Osmanağaoğlu, S. Caner Karahan, Turhan Aran, Süleyman Güven, Elif Turgut, Ahmet Menteşe, Hasan Bozkaya

**Affiliations:** ^1^Department of Obstetrics and Gynecology, Faculty of Medicine, Karadeniz Technical University, 61080 Trabzon, Turkey; ^2^Department of Biochemistry, Faculty of Medicine, Karadeniz Technical University, 61080 Trabzon, Turkey

## Abstract

*Objective*. To investigate serum levels of free *β*-HCG, progesterone, and ischemia-modified albumin (IMA) and their combined use in the prediction of first trimester abortions. *Methods*. A total of 156 pregnant women between 5 and 13 weeks of gestational age were included in this study. At admission, serum levels of free *β*-HCG, progesterone, and IMA were noted and all cases were divided into two groups; Group I (*n* = 77) resulted in abortion including missed abortion, incomplete/complete abortion, and inevitable abortion whereas Group II (*n* = 79) included normal pregnancies. *Results*. Compared to Group II, the significantly decreased value of free *β*-HCG progesterone and significantly increased value of IMA were found in Group I (*P* < 0.01, *P* < 0.01, *P* < 0.01, resp.). When combining all three parameters, sensitivity 75%, specificity 99%, PPV 98%, and NPV 76% were obtained. The multivariate logistic regression analysis revealed the free *β*-HCG, progesterone, and IMA independent factors in the prediction of abortions. *Conclusions*. The combined use of free *β*-HCG, progesterone, and IMA levels can be useful in the prediction of first trimester spontaneous abortions.

## 1. Introduction

Abortion is defined as the expulsion of the accessories of the embryo or those of fetus weighing less than 500 from the uterus cavity before the twentieth week of the pregnancy. It has been clearly shown that *β*-HCG and progesterone have a prognostic value in differentiating spontaneous abortions from the normal pregnancy.

Ischemia-modified albumin (IMA), a modification of human serum albumin caused by ischemia, measured by the albumin cobalt test has been proposed as a serum biomarker of myocardial ischemia. It is a nonspecific marker for ischemia because oxidative free radicals can be formed in every kind of ischemia. Serum IMA appears to be elevated in normal pregnancy [1, 2]. Previous reports have documented ongoing ischemia and formation of oxygen free radicals during physiological pregnancy [3–5]. Several studies were also performed to investigate IMA levels in complicated pregnancies such as preeclampsia, intrauterine growth restriction (IUGR), and recurrent pregnancy loss [6, 7] and serum IMA levels from women with preeclampsia as a consequence of defective endovascular trophoblast invasion were detected to be higher than those with normal placentation in first trimester [6] and serum IMA levels were significantly higher in women with recurrent pregnancy loss as an abnormally high hypoxic intrauterine environment which may be associated with abnormal placental development [7].

Our previous study [8] including free *β*-HCG, progesterone, and CA-125 showed that CA-125 levels are not an effective marker either alone or by adding *β*-HCG with progesterone in detecting abortions and the single measurement of free *β*-HCG or progesterone levels can be useful in the prediction of first trimester spontaneous abortions. By excluding CA-125, our aim was to investigate serum levels of free *β*-HCG, progesterone, and IMA alone and their combined use in the prediction of first trimester abortion in the present study.

## 2. Materials and Methods

The study protocol was approved by the Medical Ethics Committee of our university and a written informed consent was obtained from each patient who participated in this study. This work was supported by the Research Fund of Karadeniz Technical University, Project number: 2009.114.002.6. A total of 156 singleton pregnant women who were admitted to our department of obstetrics and gynecology between the 5th and 13th week of gestational age were included in this case-control study. Pregnant women with multiple pregnancy, ectopic pregnancy, missed abortion, blighted ovum, threatened abortion, an abnormal serum albumin level (normal level, 3–5.5 mg/dL), prior treatment with progesterone, who were smokers or had diabetes mellitus, renal, trophoblastic and thrombophilia diseases were excluded. At admission, serum levels of free *β*-HCG, progesterone, and IMA were noted and all pregnant women were followed until delivery. Gestational ages were determined by last menstrual period and the results of first trimester or early second trimester ultrasonographic examinations followed by a detailed examination at 20 weeks of gestation. Patients with known structural or chromosomal anomalies were excluded from the study. The diagnoses of inevitable, incomplete, complete, and missed abortion were made according to the criteria in the abortion classification. All cases were divided into two groups, Group I (*n* = 77) resulted with abortion including missed abortion, abortus incompletus/completus, and inevitable whereas Group II (*n* = 79) included normal pregnancies where their pregnancy did not result in abortion.

## 3. Biochemical Analyses

Venous blood samples were collected at admission from the brachial vein of the patients and centrifuged at 3000 rpm during 10 minutes in the Biochemistry Laboratory of Karadeniz Technical University. The levels of human serum *β*-HCG were determined by using Roch Hitachi Modular E-170 autoanalyzer (Tokyo, Japan) in clinical laboratory of Karadeniz Technical University Faculty of Medicine, Farabi Hospital. The results were expressed as mIU/mL. The detection range of the parameter is 0.1–10000 mIU/mL. The levels of human serum progesterone were determined by using Roch Hitachi Modular E-170 autoanalyzer (Tokyo, Japan) in clinical laboratory of Karadeniz Technical University Faculty of Medicine, Farabi Hospital. The results were expressed as ng/mL. The detection range of the parameter is 0.03–60 ng/mL. Reduced cobalt to albumin binding capacity (IMA level) was analyzed using the rapid and colorimetric method of Bar-Or et al. [9]. Two hundred *μ*L of patient serum was placed into glass tubes and 50 *μ*L of 0.1% cobalt chloride (Sigma, CoCl_2_ · 6H_2_O) in H_2_O was added. After gentle shaking, the solution was left for 10 minutes, in order to ensure sufficient cobalt albumin binding. Fifty microliters of dithiothreitol (DTT) (Sigma, 1.5 mg/mL H_2_O) was added as a colorizing agent and the reaction was quenched 2 min later by adding 1.0 mL of 0.9% NaCl. A colorimetric control was prepared for preoperative and postoperative serum samples. For the colorimetric control samples, 50 *μ*L of distilled water was substituted for 50 *μ*L of 1.5 mg/mL DTT. Specimen absorbencies were analyzed at 470 nm by a spectrophotometer (Shimadzu UV1601, Australia). The color of the DTT containing specimens was compared with that of the colorimetric control tubes. The results were reported as absorbance units (ABSUs).

The statistical Package Program for the Social Sciences (SPSS 11.0; SPSS Inc., Chicago, IL) was used for statistical analysis. Data are presented as incidence or mean ± SD. The compatibility of the data obtained by measurement of the normal distribution was studied by the Kolmogorov Smirnov test. Statistical comparisons between groups were performed using the Mann-Whitney *U* test and *χ* ^2^ test and Fisher exact test. An overall analysis used a cut-off *P* ≤ 0.05, significant. A multivariate logistic regression analysis was performed to evaluate the influence of variables on abortion according to two groups. Cut-off values were determined using receiver operating characteristic (ROC) curve analysis, and they were ≤19.98 mIU/mL for *β*-HCG (sensitivity 87.01 [77.4–93.6 95% CI]; specificity 79.75 [69.2–88 95% CI]), ≤20.45 ng/mL for progesterone (sensitivity 96.10 [89–99.2 95% CI]; specificity 74.68 [63.6–83.8 95% CI]), and >0.886 for IMA (sensitivity 75.32 [64.2–84.4 95% CI]; specificity 91.14 [82.6–96.4 95% CI]). Sensitivity and specificity were calculated according to the formulas [TP/TP + FN] × 100, [TN/TN + FP] × 100, respectively, and expressed as a percentage. In the formulas we have the following: TP: true positive; FN: false negative; TN: true negative; and FP: false positive cases.

## 4. Results

The study group consited of 77 pregnant women where their pregnancy resulted in abortion. The control normal pregnant group consisted of 79 normal pregnancies. Normal pregnancy was defined as delivery after 37 weeks of gestation where the neonatal birthweight was above the 5th centile for gestation [10].

The general characteristics of cases during the admission and the mean values of *β*-HCG, progesterone, and IMA for each group are represented in Table 1. No difference was detected between the two groups in terms of age, number of pregnancy, mean pregnancy weeks, and number of previous abortions (*P* > 0.05, *P* > 0.05, *P* > 0.05, and *P* > 0.05, resp.). When the groups were compared, a statistical difference was found in terms of mean *β*-HCG, progesterone, and IMA values (*P* < 0.01, *P* < 0.01, and *P* < 0.01, resp.), (Table 2).

The sensitivity, specificity, and positive predictive and negative predictive values are given in Table 3. When 20 mIU/mL was taken as cut-off value for *β*-HCG level, a significant difference was detected between both groups (*P* < 0.01). When 20.45 ng/mL was taken as cut-off value for progesterone level, a statistically significant difference was detected markedly between both groups (*P* < 0.01). When 0.886 was taken as cut-off value for IMA level, a significant difference was detected between the groups (*P* < 0.05) (Table 3). The sensitivity, specificity, and positive and negative indicative values in determining abortion when all parameters are used together are given in (Table 3). By taking the age as constant in multivariate logistic regression analysis it was observed that *β*-HCG, progesterone, and IMA are independent determining factor in predicting abortion (Table 4).

## 5. Discussion

In aggrement with the previous studies [11, 12], we found *β*-hCG levels significantly lower in the group where their pregnancy resulted in abortion than that of the group where no abortion was detected. In the previous study, when the healthy pregnancy group as control was compared with the abortus group, we found sensitivity as 91%, specificity as 82%, and positive predictive value as 46% in abortion cases when we took the estimation value as <20 mIU/mL for *β*-HCG. The sensitivity value of the single measurement of *β*-HCG (≤20 mIU/mL) and the sensitivity of using *β*-HCG and progesterone together that we obtained as 88% and the specificity of both as 80% in detecting abortions are consistent with those of our previously reported study [8] and those found in the study of Daily et al. [13]. In addition, similar to our previously reported study, we detected that *β*-HCG has an independent effect on abortion in the multiple regression analysis in the present study. Daily et al. [13] found the sensitivity of progesterone as 75% and specificity as 78% when they took the estimation value as <15 ng/mL in the differentiation of the abnormal pregnancy and the normal pregnancy. Similarly, according to the study of Daily et al. [13], we found previously the sensitivity of progesterone as 91% and the specificity as 89% in the differentiation of pregnancies resulting in abortion from normal pregnancies when we took the estimation value as <15 ng/mL in pregnancies between the 5th and 13th week of gestational age [8]. In the present study, we found the sensitivity as 96% and the specificity as 75% in the detection of abortion when we took the estimation value as ≤20.45 ng/mL. Our results are consistent with those of Daily et al. and Hahlin et al. [13, 14] and those found in our previously reported study [8]. It is clear that when the cut-off level of progesterone will be found higher, the detection of spontaneous abortion rate will increase. In addition, similar to our previously reported study, we detected that *β*-HCG has an independent effect on abortion in the multiple regression analysis in the present study. Furthermore, according to our previously reported study, we detected similarly that the use of both *β*-HCG and progesterone together instead of studying *β*-HCG and progesterone once does not provide additional benefit in terms of increasing the sensitivity value IMA; a protein elevated in cardiac ischemia, is also increased to supraphysiological levels in early normal pregnancy. This finding supports the hypothesis that normal trophoblast development is stimulated by a hypoxic intrauterine environment and abnormally high intrauterine hypoxia and subsequent reperfusion oxidative damage may be associated with defective trophoblast development [5]. However, the mechanism of IMA related pregnancy is not totally understood and various studies have demonstrated.that IMA may increase in normal pregnancy [1, 2], remain unchanged [1] or increase [3–5] in pregnancy with preeclampsia. It can be also speculated that serum IMA levels from women with ectopic pregnancy may be detected to be higher than those with normal placentation in first trimester by a hypoxic environment and reperfusion oxidative damage. Differences in methodology or specificity of procedures to determine the IMA and the differences of cut-off value have probably resulted in differences outcome. In addition, the types of abortus vary among studies; therefore the heterogeneity in classification may differ the outcome. The test combinations and the timing of screening during pregnancy may also influence the different outcome. We found that IMA levels were significantly higher in the abortus groups than in the control group and IMA with a cut-off point of 0.886 identified women with abortus with sensitivity 74% and specificity 91%. In the study of Hjortshøj et al. [15] the sensitivity of admission IMA (at optimal cut-off threshold −91 U/mL) for a final diagnosis of acute coronary syndrome was 86%, specificity 49%, and NPV 88%. This rise in IMA may be a clinical manifestation of abnormally high intrauterine hypoxia and subsequent reperfusion oxidative damage that underlie defective endovascular trophoblast development. First trimester serum IMA may thus be a potential biomarker for abnormal placental development.

In conclusion, based on the findings that the single measurement of IMA had higher specificity and PPV, the single and the combination measurements of free *β*-HCG and progesterone levels had higher sensitivity in the present study and as independent variables the combined use of free *β*-HCG, progesterone, and IMA levels can be useful in the prediction of first trimester spontaneous abortions.

## Figures and Tables

**Table 1:** Baseline characteristics of study group during the admission.

	Group 1 (*n* = 77)	Range	Group 2 (*n* = 79)	Range	*P*
Maternal age (years)	28 ± 5.8		27 ± 5.1		>0.05^b^
Gravidity (*n*)	2 ± 1.4		2 ± 1.3		>0.05^b^
Gestation at presentation (weeks)	8.9 ± 2.2		9.3 ± 2.0		>0.05^b^
Number of previous miscarriages (*n*)	0 ± 0.7		0 ± 0.6		>0.05^b^
Free *β*-HCG (mIU/mL)	13.0 ± 35.1	158.420	38.9 ± 48.6	225.156	<0.01^a∗^
Progesterone (ng/mL)	12.3 ± 8.0	54.27	26.7 ± 11.9	70.54	<0.01^a∗^
Ischemia-modified albumin	0.80 ± 0.2	0.706	0.64 ± 0.1	0.765	<0.01^a∗^

Data are presented as mean ± SD.

^a^Mann-Whitney *U* test was used.

^b^Student's *t*-test was used.

∗Significant.

**Table 2:** The comparison of free *β*-HCG, progesterone and ischemia-modified albumin (IMA) values upon their estimation values.

	Group 1 (*n* = 77)		Group 2 (*n* = 79)		*P*
	*n*	(%)	*n*	(%)	
Free *β*-HCG ≤20 mIU/mL	68	88	16	20	<0.01∗
Free *β*-HCG >20 mIU/mL	9	12	63	80	
Progesterone ≤20.45 ng/mL	74	96	20	25	<0.01∗
Progesterone >20.45 ng/mL	3	4	59	75	
IMA <0.886	19	25	72	91	<0.01∗
IMA >0.886	58	75	7	9	

^*^Significant.

**Table 3:** The sensitivity value in determining abortion when free *β*-HCG (≤20 mIU/mL), progesterone (≤20.45 ng/mL), and ischemia-modified albumin (IMA) (>0.886) levels are used as single or combined parameters.

	Sensitivity (%)	Specificity (%)	PPV (%)	NPV (%)
Free *β*-HCG	87	80	86	81
Progesterone	96	75	95	79
IMA	74	91	77	95
Free *β*-HCG (≤20 mIU/mL) + progesterone (≤20.45 ng/mL)	88	80	81	87
Free *β*-HCG (≤20 mIU/mL) + IMA (>0.886)	75	99	98	80
Progesterone (≤20.45 ng/mL) + IMA (>0.886)	75	97	97	80
Free *β*-HCG (≤20 mIU/mL) + progesterone (≤20.45 ng/mL) + IMA (>0.886)	75	99	98	76

PPV: positive predictive value.

NPV: negative predictive value.

**Table 4:** Outcome of multivariate logistic regression analysis in cases with free *β*-HCG, progesterone, and ischemia-modified albumin (IMA).

Dependent variable: abortus	Statistical significance	RR	95% CI
Free *β*-HCG	*P* = 0.001	1.00	1.00-1.00
Progesterone	*P* = 0.000	0.80	0.74–0.86
IMA	*P* = 0.000	78.92	8.10–768.82
Age	*P* = 0.152	1.06	0.97–1.15

RR: relative risk.

CI: confidence interval.
